# Preparation of Cationic MOFs with Mobile Anions by Anion Stripping to Remove 2,4-D from Water

**DOI:** 10.3390/ma10080879

**Published:** 2017-07-30

**Authors:** Tao Chen, Cong Zhang, Yuemei Qin, Haiguan Yang, Peng Zhang, Fanggui Ye

**Affiliations:** State Key Laboratory for the Chemistry and Molecular Engineering of Medicinal Resources, College of Chemistry and Pharmaceutical Science of Guangxi Normal University, Guilin 541004, China; chenmaoen2015@163.com (T.C.); congzhangchem@163.com (C.Z.); qym0774@163.com (Y.Q.); yanghaiguan135588@163.com (H.Y.); 15677330721@163.com (P.Z.)

**Keywords:** adsorption, 2,4-dichlorophenoxyacetic acid, ion exchange, metal-organic frameworks

## Abstract

A cationic porous framework with mobile anions (MIL-101(Cr)-Cl) was easily and successfully synthesized by utilizing the stronger affinity of F^−^ to Al^3+^ than Cr^3+^ in the charge-balanced framework of MIL-101(Cr). The structure, morphology and porosity of MIL-101(Cr)-Cl were characterized. The obtained new materials retain the high surface area, good thermostability, and structure topology of MIL-101(Cr). With the mobile Cl^−^ anion, MIL-101(Cr)-Cl can be used as an ion-exchange material for anionic organic pollutions. In this work, 2,4-dichlorophenoxyacetic acid (2,4-D) was used as a model to test the absorption performance of this new material. This new material exhibited improved adsorbability compared to that of the original metal-organic frameworks (MOFs). At the same time, this material also shows high anti-interference performance with changing solution pH.

## 1. Introduction

Water pollution has become a global problem and is receiving significant attention, especially in developing countries. Pollutants exert a detrimental effect on the environment and human health, and hence, many research efforts have focused on removing pollutants from waste water. So far, various technologies have been used to remove pollutants, including photo-reduction or degradation [1], bio-degradation [2], ion exchange [3] and adsorption [4]. Among the technologies, ion exchange is one of the most eco-friendly and economical technologies, because most of the materials can be reused, this method has high sensitivity and selectivity, and the operation process is simple. The existing organic materials that can be used in ion exchange are sol–gel adsorbents [5] and polymer resins or membranes [6], which show rapid exchange kinetics and high ion-exchange capacity, but their low thermal and chemical stability limit their application in water purification. Although inorganic ion exchangers such as zeolites [7] and layered double hydroxides [8] have high thermal and chemical stability, their exchange kinetics and ion-exchange capacity are low. Therefore, developing new materials with high ion-exchange capacity and thermal and chemical stability is still a challenging task.

Metal-organic frameworks (MOFs), which are formed by using a pre-formed organic linker with a metal ion or cluster, are a class of supramolecular material. Owing to their unique structure and properties such as coordination with unsaturated metal sites, good thermal stability, high surface area, and adjustable pore size, as well as their ability to modify their pore surfaces, MOFs have been widely used in gas storage [9] and separation [10], catalysis [11], drug delivery [12], molecular sensing [13], and photoelectron chemistry and electro-chemistry [14]. MOFs have also been used for removing various hazardous substances from the environment. The adsorption mechanisms of most hazardous substances are based on acid-base interactions, π-complexation, H-bonding, and coordination with open metal sites. However, the adsorption capacity may be affected by operating conditions such as the pH or ionic strength. Some studies on charged MOFs materials [15,16,17,18] have been reported and some of these materials were used as adsorbents to remove pollutants from waste water using ion exchange.

2,4-Dichlorophenoxyacetic acid (2,4-D) was one of the most widely used acidic herbicides and plant growth regulators in the past three decades. However, it is considered to be a potential carcinogenic and teratogenic pollutant. Its low pKa (2.8) and large-scale application led to its accumulation in soils and environmental waters [19]. Therefore, eliminating 2,4-D from the environment is a necessary goal. So far, various technologies have been used for removing 2,4-D, including ozonation, adsorption, ion exchange, electrochemical technology, and biological processes [20,21,22,23,24]. Adsorption and ion exchange are often used to treat 2,4-D-contaminated water, but both have their own strengths and weaknesses. Jhung et al. [25] studied MOFs (MIL-53(Cr)) for the adsorptive removal of 2,4-D from water for the first time and with a high absorption capacity, but the adsorption capacity decreased with increasing pH and negligible adsorption was observed when pH > 6. Bhardwaj et al. [26] demonstrated that surfactant-modified tectosilicates (clinoptilolite and zeolite Y) and phyllosilicates (bentonite and montmorillonite) can be used to remove 2,4-D from water based on ion exchange, although the adsorption capacities are lower and the adsorption capacity drops off slowly at high pH.

In the present work, a cationic MOF (denoted as MIL-101(Cr)-Cl) was prepared by sequentially exchanging F^−^ in original MIL-101(Cr) with Cl^−^. This preparation method is simple and does not require a cumbersome modification process. We can obtain this ion-exchange material, which has excellent performance by a simple hot AlCl_3_ solution treatment step. In addition, this cationic MOF has higher adsorptive capacity for 2,4-D than other materials, even at high pH.

## 2. Results and Discussion

### 2.1. Characterization of As-Prepared Materials

**Figure 1:**
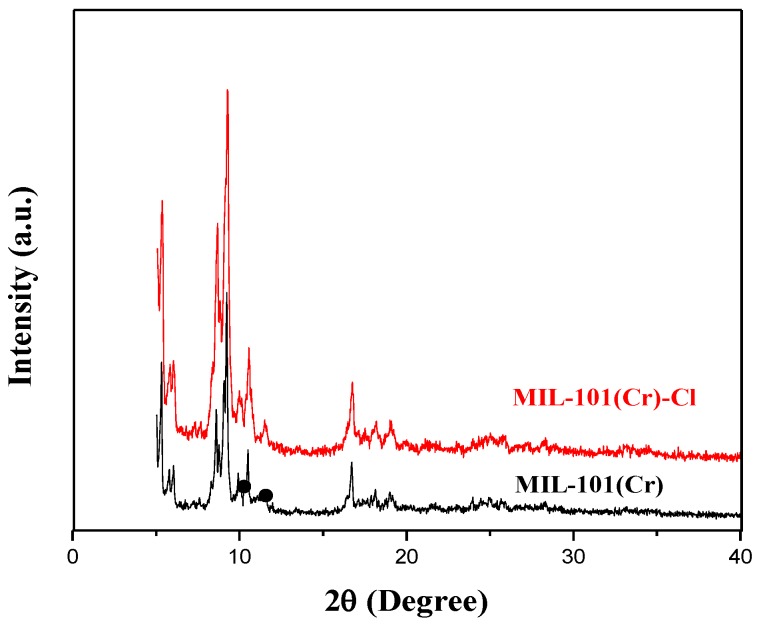
Powder X-ray diffraction (PXRD) patterns of MIL-101(Cr) and MIL-101(Cr)-Cl.

The crystallinity of MIL-101(Cr) and MIL-101(Cr)-Cl were monitored by powder X-ray diffraction (PXRD). As indicated by the PXRD (Figure 1), the synthesized MIL-101(Cr) exhibited the same characteristic structure in consistence as the reported work [12]. After anion stripping, the material (MIL-101(Cr)-Cl) still remained highly crystalline. It means that MIL-101(Cr)-Cl may still retain some characteristics of MIL-101(Cr), such as porosity.

**Figure 2:**
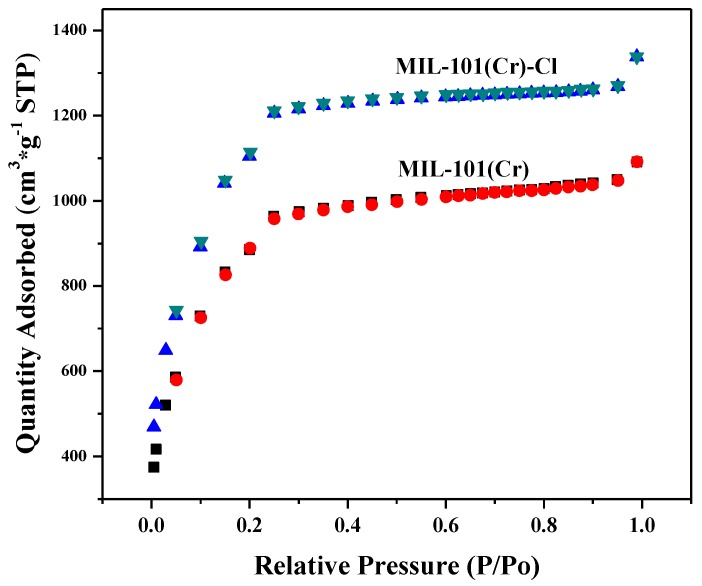
N_2_ adsorption–desorption isotherms of as-synthesized MIL-101(Cr) and MIL-101(Cr)-Cl.

To further characterize the porosity of the as-prepared materials, they were examined by nitrogen sorption. As shown in Figure 2, both samples have type-I isotherms with hysteresis loops, indicating a mesoporous structure. The N_2_ uptake of MIL-101(Cr)-Cl was much higher than that of the pretreated original MIL-101(Cr). The Brunauer-Emmett-Teller (BET) surface areas, pore volumes, and apertures are summarized in Table 1, and the pore size distribution is shown in Figures S1 and S2. The BET surface area and the pore volumes became larger after anion stripping. During the anion stripping procedure, H_2_BDC residues within the pores of MIL-101(Cr) were stripped of F^−^, and it is the reason why the BET surface area and the pore volumes became larger. The average pore size—which was the same as in the reported work [27]—remained the same, suggesting that the microvoids in MOFs did not collapse after anion stripping. 

**Figure 3:**
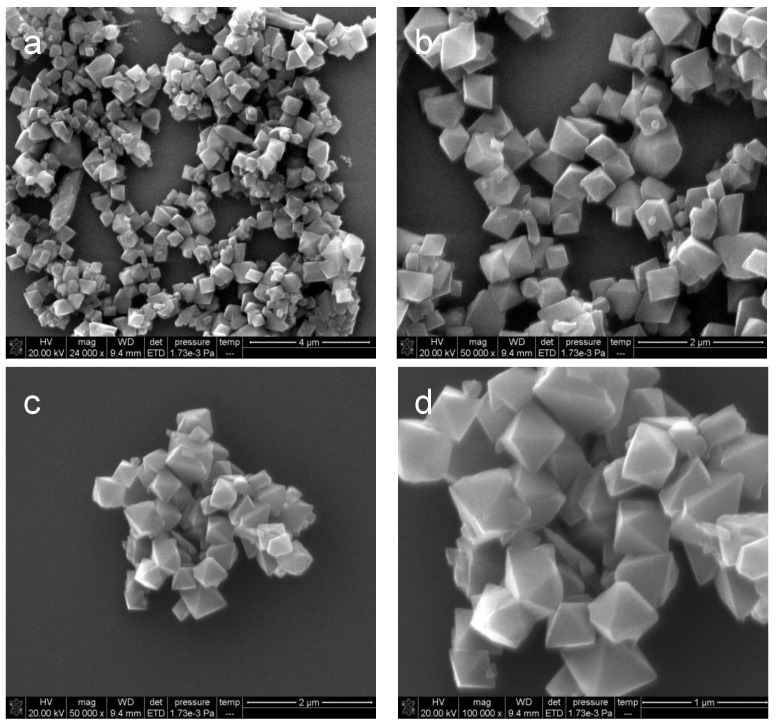
SEM images of MIL-101(Cr) (**a**,**b**) and MIL-101(Cr)-Cl (**c**,**d**).

**Figure 4:**
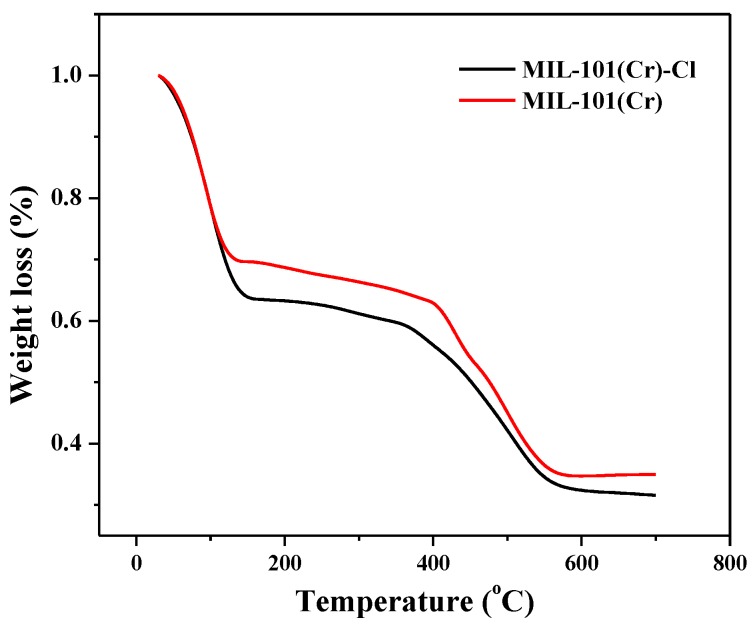
TGA graph of MIL-101(Cr) (red line) and MIL-101(Cr)-Cl (black line).

The morphologies of MIL-101(Cr) and MIL-101(Cr)-Cl were investigated by Field Emission Scanning Electron Microscopy (SEM) (Figure 3). From Figure 3, it was apparent that the morphologies and size changed little after anion stripping. The sizes of these two materials were distributed homogeneously. This means that the SEM results agreed with the PXRD patterns and porosity results. Also, the Energy Dispersive Spectro-scopy (EDX) date showed that the Cl^−^ content of MIL-101(Cr)-Cl was 10.58 wt % (Table S1). The Thermo Gravimetric Analyzer (TGA) curves of MIL-101(Cr) and MIL-101(Cr)-Cl were obtained in air. As shown in Figure 4, the as-prepared materials had two weight loss steps. The first step was due to dehydrate of water that adsorbed within the pores of MIL-101(Cr), and the second step indicated that MIL-101(Cr) is stable up to 400 °C. Therefore, the materials we prepared were typical MIL-101(Cr), and changed little after anion stripping. All the characterizations proved that MIL-101(Cr)-Cl retains the performance of MIL-101(Cr).

To demonstrate that Cl^−^ has taken the place of F^−^ and that Cl^−^ can be removed, we performed some simple experiments [28]. One is the precipitation experiment performed before and after ion-exchange with NaNO_3_. Typically, 5 mg of MIL-101(Cr)-Cl powders was soaked in 10 mL water or 80 mg/mL NaNO_3_ for about 12 h. After centrifuging, the upper liquid was taken out, and then 5 mM AgNO_3_ solution was added. 

**Figure 5:**
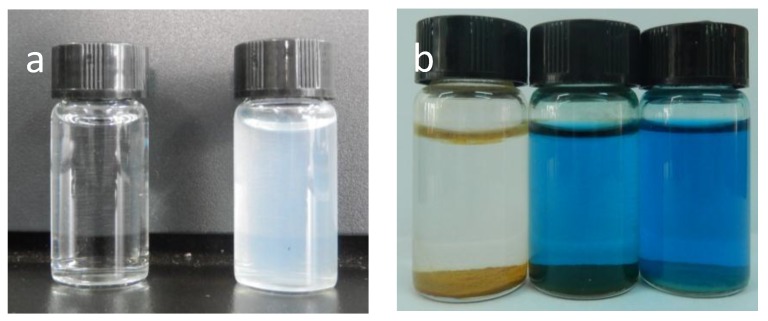
(**a**) Left is the supernatant liquid of MIL-101(Cr)-Cl soaked in water with added AgNO_3_ solution, right is the supernatant liquid of MIL-101(Cr)-Cl soaked in NaNO_3_ solution with added AgNO_3_ solution; (**b**) Left is methyl orange (MO) after adsorption by MIL-101(Cr)-Cl, middle is MO + MB after adsorption by MIL-101(Cr)-Cl, and right is MB after adsorption by MIL-101(Cr)-Cl.

From Figure 5a, we can see that a white precipitate was found in the sample soaked in 80 mg/mL NaNO_3_, which was not found in the sample soaked in water. This phenomenon means that Cl^−^ has taken the place of F^−^ and Cl^−^ can be removed. The mobility of Cl^−^ was further confirmed by adsorbing anionic dye molecules under the same condition. Typically, 10 mg of MIL-101(Cr)-Cl powder was added into a 10 mL vial, and 10 mL of 50 mg/L methyl orange (MO), methylene blue, or mixed dye was added to the vial. After 4 h of adsorbing and standing, it is clear that MIL-101(Cr)-Cl is more receptive to the negatively charged MO (Figure 5b).

### 2.2. Adsorptive Kinetics

**Figure 6:**
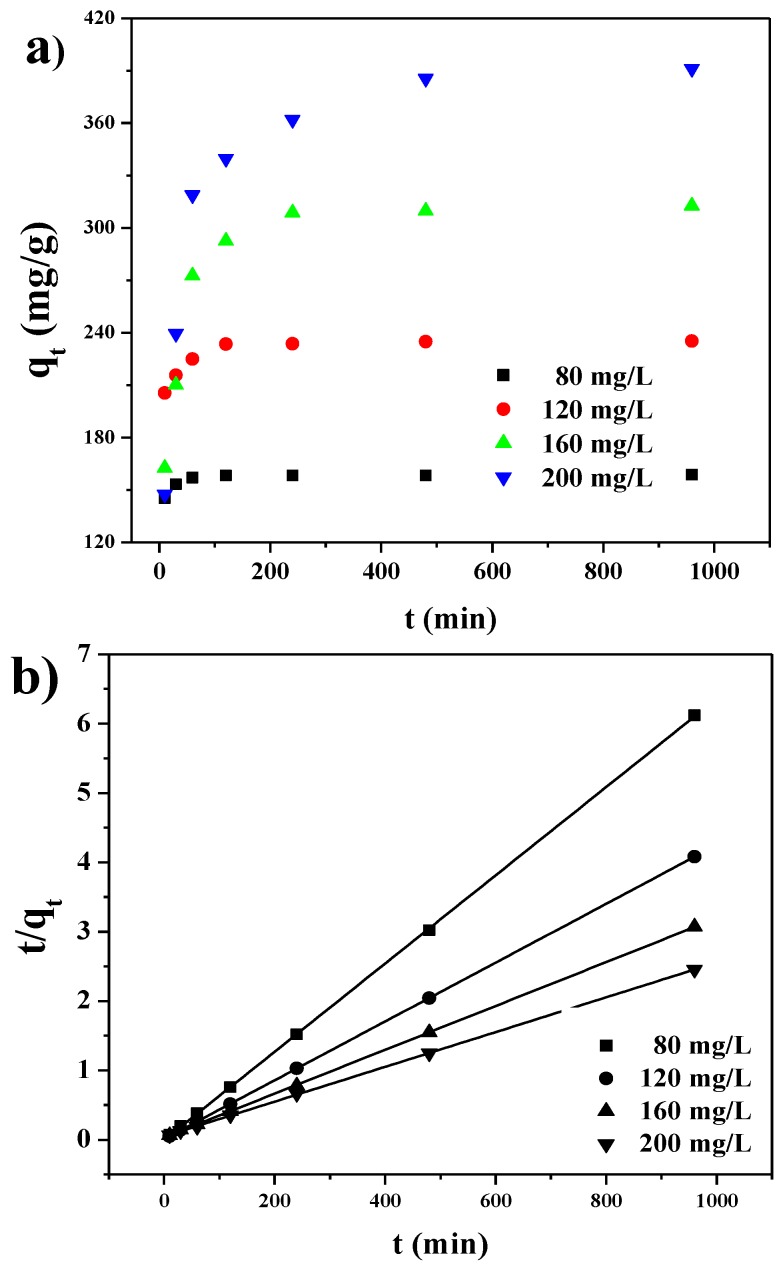
(**a**) Effect of contact time on the adsorption of 2,4-D on MIL-101(Cr)-Cl (10 mg) at different initial concentrations of 2,4-D at 30 °C; (**b**) Plots of pseudo-second-order kinetics for the adsorption of 2,4-D at 30 °C.

The kinetics of the adsorption of 2,4-D from water onto MIL-101(Cr)-Cl were studied as a function of contact time-dependent adsorption capacity. From Figure 6a, we can see that the adsorption capacities increased rapidly in the first 100 min and then slowed down; adsorption equilibrium was reached in about 200 min. The rapid removal at the initial time could be attributed to the abundance of Cl^−^ sites that can be removed in the MOFs and the positively charged surface of MIL-101(Cr)-Cl. With time, increasing concentration of Cl^−^ sites and reduction in the amount of the positive charge, the adsorption rate decreased gradually at high conversion. Besides, the 2,4-D adsorbed in the MOFs at the initial time might partially block the cavities inside the MOFs, which has a negative impact on the adsorption rate. 

The adsorption kinetics were simulated using a pseudo-second-order model. The pseudo-second-order equations can be expressed as:

(1) $$\frac{dq}{dt}=k_{2}{\left(q_{e}-q_{t}\right)}^{2},$$

or

(2) $$\frac{t}{q_{t}}=\frac{1}{k_{2}q_{e}{}^{2}}+\frac{t}{q_{e}}$$

where *q_e_* (mg/g) and *q_t_* (mg/g) are the 2,4-D adsorbing capacity at equilibrium and the time *t* (min), respectively; *k*_2_ (g/(mg min)) is the pseudo-second-order adsorption rate constant; *k*_2_ can be calculated from the slope and intercept of the plot of *t*/*qt* versus *t* in Figure 6b. 

From Figure 6b, we can see that the correlation coefficients at different temperatures are nearly 1.00, which means that 2,4-D adsorption on MIL-101(Cr)-Cl follows the pseudo-second-order model. This model was developed based on the assumption that the limiting step may be a chemisorptions process involving valency forces via the exchange of elections between the adsorbate and adsorbent.

### 2.3. Adsorptive Isotherms

**Figure 7:**
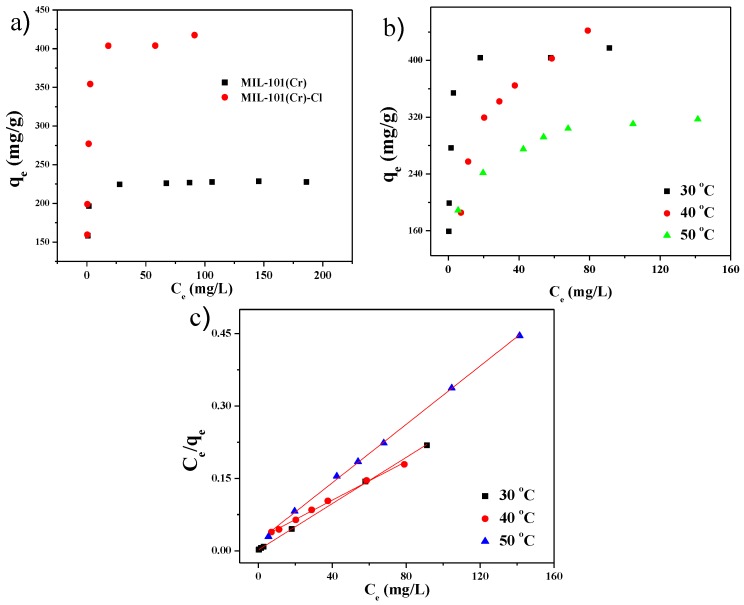
(**a**) Standard adsorption curves for 2,4-D on MIL-101(Cr) (black line) and MIL-101(Cr)-Cl (red line) at 30 °C; (**b**) adsorption isotherms for MG on MIL-101(Cr)-Cl at different temperatures; and (**c**) Langmuir plots of the isotherms for the adsorption of 2,4-D adsorption onto MIL-101(Cr)-Cl.

The adsorption isotherm model was widely used to describe the adsorption progress and investigate the mechanisms of adsorption. In this work, the adsorption isotherms of 2,4-D over MIL-101(Cr) and MIL-101(Cr)-Cl were obtained by adsorbing for ~16 h, and the results are shown in Figure 7.

From Figure 7a, it is clear that the adsorption capacities of MIL-101(Cr)-Cl were much higher than those of MIL-101(Cr) under the same test conditions, suggesting that anion stripping could enhance the efficiency of adsorbing 2,4-D.

In order to describe the adsorption isotherms of 2,4-D adsorbed over MIL-101(Cr) more clearly, the commonly used Langmuir model and three different temperatures were selected for this study [29]. The Langmuir equation can be expressed as:

(3) $$\frac{C_{e}}{q_{e}}=\frac{1}{q_{\max}K_{L}}+\frac{C_{e}}{q_{\max}}$$

or

(4) $$q_{e}=\frac{q_{\max}K_{L}C_{0}}{1+K_{L}C_{e}}$$

where *K_L_* (L/mg) is the Langmuir constant, and *q*_max_ (mg/g) is the maximum adsorption.

As is shown in Figure 7c, this test proved that the method conforms well to the Langmuir model. This means that this model can explain the adsorption of 2,4-D on MIL-101(Cr)-Cl. At the same time, we can see that the highest adsorption capacity is at 40 °C; we also calculated the adsorption capacities at 30 °C, 40 °C and 50 °C as 416.7 mg/g, 497.5 mg/g and 330.3 mg/g, respectively, from the slope of the line shown in Figure 7b.

### 2.4. Adsorption Mechanism

**Figure 8:**
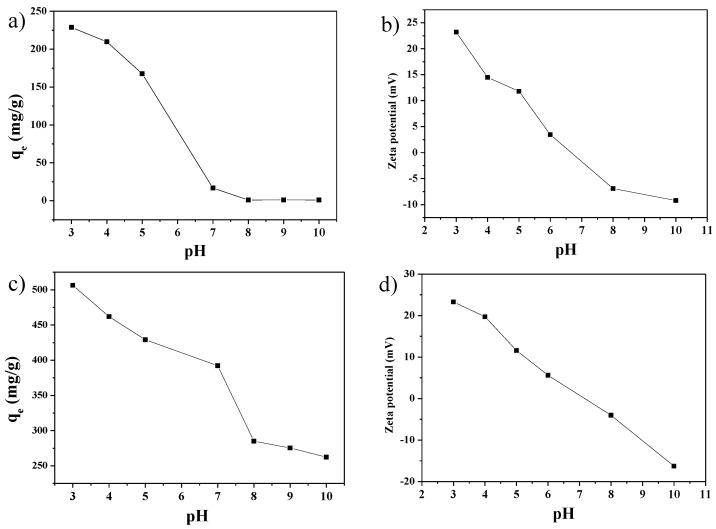
(**a**) Effect of pH of 2,4-D solution on the adsorption capacity of 2,4-D over MIL-101(Cr); (**b**) zeta potential of MIL-101(Cr) in different pH solutions; (**c**) effect of pH of 2,4-D solution on the adsorption capacity of 2,4-D over MIL-101(Cr)-Cl; (**d**) zeta potential of MIL-101(Cr)-Cl in different pH solutions.

As shown in Figure 8a, the adsorption capacity of 2,4-D on MIL-101(Cr) decreased with increasing pH. The low pKa (2.8) made it easy to ionize when the pH of solution is greater than 3.0. According to Figure 8b, the zeta potential of MIL-101(Cr) is positive when pH < 6. Hence, the repulsive electrostatic interaction between MIL-101(Cr) and 2,4-D can explain this phenomenon. The isoelectric point of MIL-101(Cr) is about 6.5, and the reason for the lower adsorption capacity was that MIL-101(Cr) and 2,4-D had positively charged surfaces. While the adsorption capacity does not reduce to zero, the lower capacity might be explained by the π–π stacking of benzene rings between 2,4-D and the organic ligands in MOFs. This result fits with previous reports [2,25,30] perfectly.

Comparing the trend of the pH and zeta potential of MIL-101(Cr) and MIL-101(Cr)-Cl (Figure 8a–d), it is obvious that their trends are similar. Hence, the capacity of MIL-101(Cr)-Cl should be the same as that of MIL-101(Cr), but from Figure 8c, it is clear that the capacity remained high when the pH > 6. This means that there must be another force between 2,4-D and adsorbents besides the electrostatic interaction and π–π stacking. Since the Cl^−^ in MIL-101(Cr)-Cl can be removed, it is speculated that ion exchange also occurs during the adsorption process. This phenomenon has also been experimentally verified (see the Supporting information); the precipitation experiment before and after the adsorption of 2,4-D also showed that MIL-101(Cr)-Cl had excellent performance in adsorbing negatively charged organic pollutants (Figure S3). The capacity of MIL-101(Cr)-Cl was much higher than that of MIL-101(Cr) at pH = 7.0, which is a visible sign of the ion-exchange adsorption process. Both tests proved that ion change existed during the adsorbing of 2,4-D on MIL-101(Cr)-Cl.

### 2.5. Regeneration

**Figure 9:**
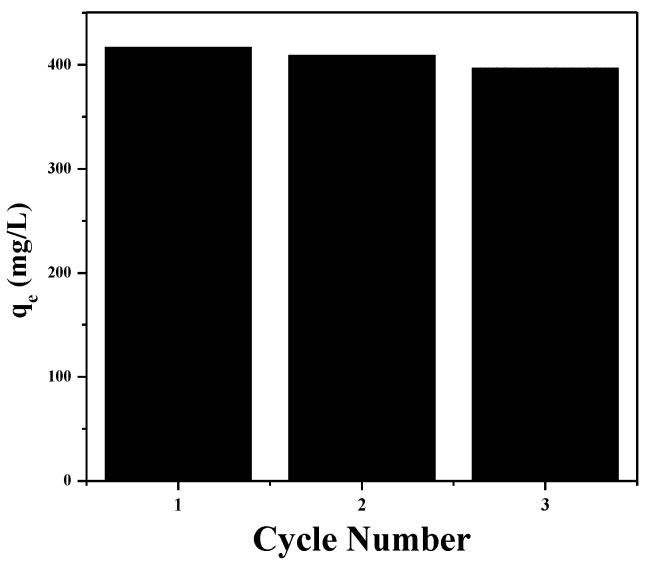
Eluting regeneration cycles and adsorption capacity.

From the economic and environmental viewpoints, it is necessary to reuse adsorbents. The desorption and regeneration of 2,4-D over MIL-101(Cr)-Cl were carried out by washing with NaCl-HCl solution (pH = 2.0) and ethanol. As can be seen from Figure 9, the adsorption capacity changed little in three cycles, which reveals that the materials can be reused.

## 3. Materials and Methods 

### 3.1. Characterization of As-Prepared Materials

Terephthalic acid (H_2_BDC) and aluminum (III) chloride hexahydrate (AlCl_3_·6H_2_O) were purchased from Aladdin (Shanghai, China). Chromium nitrate (Cr(NO_3_)_3_·9H_2_O), ammonium fluoride (NH_4_F), ethanol, and hydrofluoric acid (HF ≥ 40%) were received from Shanghai Chemical Reagents Corporation (Shanghai, China). 2,4-D was purchased from Alfa Aesar (Shanghai, China). Ultrapure water was produced with a Millipore purification system (Bedford, MA, USA). All the above chemicals were at least of analytical reagent grade, and used without any purification.

### 3.2. Apparatus

Powder X-ray diffraction analysis (PXRD) was carried out using a D/max 2550 VB/PC diffractometer (Rigaku, Tokyo, Japan) operating with Cu-Kα radiation (λ = 0.15418 nm). The morphologies of the as-synthesized samples were characterized using field emission scanning electron microscopy (SEM; NoVaTM Nano SEM 430, FEI, Hillsboro, OR, USA). The specific surface areas and pore-size distributions were calculated by Brunauer-Emmett-Teller (BET) methods measured on 3Flex (Micromeritics, Atlanta, GA, USA) by nitrogen adsorption and desorption at 77 K.

### 3.3. Synthesis of MIL-101(Cr)

**Scheme 1:**
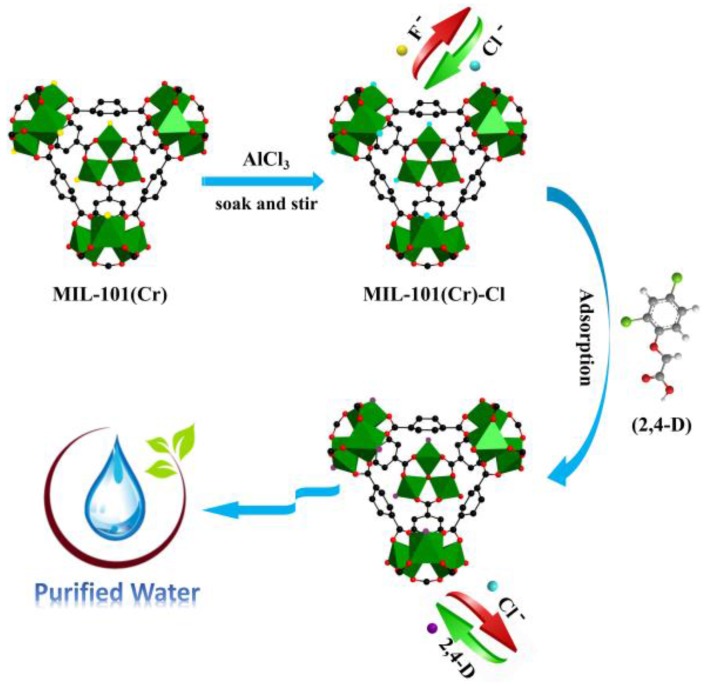
Synthesis of MIL-101(Cr)-Cl.

As illustrated in Scheme 1, MIL-101(Cr) was prepared by a hydrothermal reaction previously reported by Férey and co-workers [31]. Typically, 166 mg of H_2_BDC, 400 mg of Cr(NO_3_)_3_·9H_2_O, and 1 mmol HF were dissolved in 4.8 mL of water, and then loaded into a Teflon-lined autoclave at 220 °C for 8 h. We obtained a highly crystallized green powder (original MIL-101(Cr)). However, the unreacted Cr(III) and H_2_BDC are present both within and outside the pores of MIL-101(Cr). Therefore, it is necessary to further purify the as-synthesized MOFs. The purification method comprises the following steps: (1) the crystalline MIL-101(Cr) product was filtered after the reaction system cooled slowly to room temperature, and then washed with a large amount of hot water to remove the unreacted Cr(III) and H_2_BDC on its surface; (2) the resulting solid was soaked in 30 mmol NH_4_F solution at 80 °C, stirred for 24 h in order to swap out the unreacted Cr(III) and H_2_BDC within the pores of MIL-101(Cr), and immediately filtered; (3) the residue was washed with a certain amount of water and ethanol. The solid was finally dried at 150 °C for 24 h under vacuum.

### 3.4. Anion Stripping of MIL-101(Cr)

To the as-synthesized MIL-101(Cr) (50 mg) in a 100 mL vial was added 50 mL 30 mmol of AlCl_3_ solution, and then the vial was shifted into a 90 °C oven and kept for about ~16 h. The final material (denoted MIL-101(Cr)-Cl) was obtained by centrifugation and washed with water until the effluents did not turn AgNO_3_ turbid. The as-prepared material was degassed under vacuum at 150 °C overnight before use.

### 3.5. Adsorption Procedure

The working solution of 2,4-D used in this test was prepared by the dilution of a stock solution (300 mg/L), which was prepared by dissolving powdered 2,4-D in deionized water. Before adsorption, the as-prepared materials, MIL-101(Cr) and MIL-101(Cr)-Cl, were degassed and dried under vacuum at 150 °C overnight. To an exact amount of degassed and dried as-prepared materials (5 mg) placed in a 20 mL vessel, was added 10 mL of 2,4-D solution. The vessel was fixed on a vortex generator and placed into a water bath. After a pre-determined adsorption time, the suspensions were separated using a syringe filter. The concentrations of 2,4-D in the solution were determined using a spectrophotometer (Agilent, Shanghai, China) at 283 nm after taking the UV spectra of the solution. The standard curve of 2,4-D (Figure S4) was obtained from standard solutions (5–200 mg/L) at pH = 3.5.

The adsorption capacity at equilibrium was calculated as follows:

(5) $$q_{e}=\frac{C_{0}-C_{e}}{M}V,$$

where *q_e_* (mg/g) is the equilibrium adsorption capacity of 2,4-D on the as-prepared material; *C*_0_ (mg/L) and *C_e_* (mg/L) are the initial and equilibrium concentrations of 2,4-D, respectively; *V* is the volume of 2,4-D solution (L); and *M* is the weight of the as-prepared material (g).

## 4. Conclusions

In summary, we successfully synthesized MIL-101(Cr) and MIL-101(Cr)-Cl by a simple hot AlCl_3_ solution treatment step. Both of them were used as adsorbents for removing 2,4-D. The experimental results show that after modification, MIL-101(Cr) has a higher capacity and the capacity remained high at high pH. While studying the adsorption mechanism, we found that in addition to electrostatic interaction and π–π stacking, ion exchange might also play a large part in the adsorption process. Hence, MIL-101(Cr)-Cl may have a great potential in adsorbing negatively charged organic pollutants, and this method of exchanging F^−^ with Cl^−^ in the original MOFs could be generalized to generate new charged MOFs.

## Figures and Tables

**Table 1:** Some properties of MIL-101(Cr) and MIL-101(Cr)-Cl for N_2_ adsorption experiments at 77 K.

Sample	S_BET_^a^ (m^2^/g)	V_p_^b^ (cm^3^/g)	D_p_^c^ (nm)
MIL-101(Cr)	3133.7	1.688	3.06
MIL-101(Cr)-Cl	3931.8	2.070	3.06

^a^ Brunauer-Emmett-Teller (BET) surface area; ^b^ total pore volume; ^c^ average pore diameter.

## Supplementary Materials

The following are available online at www.mdpi.com/1996-1944/10/8/879/s1, Figure S1: (a) The BJH pore size of MIL-101(Cr); (b) The HK pore size of MIL-101(Cr). Figure S2: (a) The BJH pore size of MIL-101(Cr)-Cl; (b) The HK pore size of MIL-101(Cr)-Cl. Figure S3: The photos of (a) 100 mg/L of 2,4-D added AgNO_3_ solution; (b) after adsorbed 2,4-D, the supernatants added AgNO_3_ solution. Figure S4: The calibration curve of 2,4-D, A = 0.0085C + 0.0184, R^2^ = 0.999. Table S1: The fluorine and chlorine content in MIL-100-Cr and MIL-100-Cr-Cl.
