# Supplementary material for: Preparation of Cationic MOFs with Mobile Anions by Anion Stripping to Remove 2,4-D from Water
Source: Materials (Basel). 2017 Jul 30;10(8):879. doi: 10.3390/ma10080879 (PMC5578245; doi:10.3390/ma10080879)
Supplement: Supplementary file 1 [file materials-10-00879-s001.pdf]

Supporting information:

Preparation of cationic MOFs with mobile anions by anion stripping to remove 2,4-D from water

Tao Chen, Cong Zhang, Yuemei Qin, Haiguan Yang, Peng Zhang and Fanggui Ye*

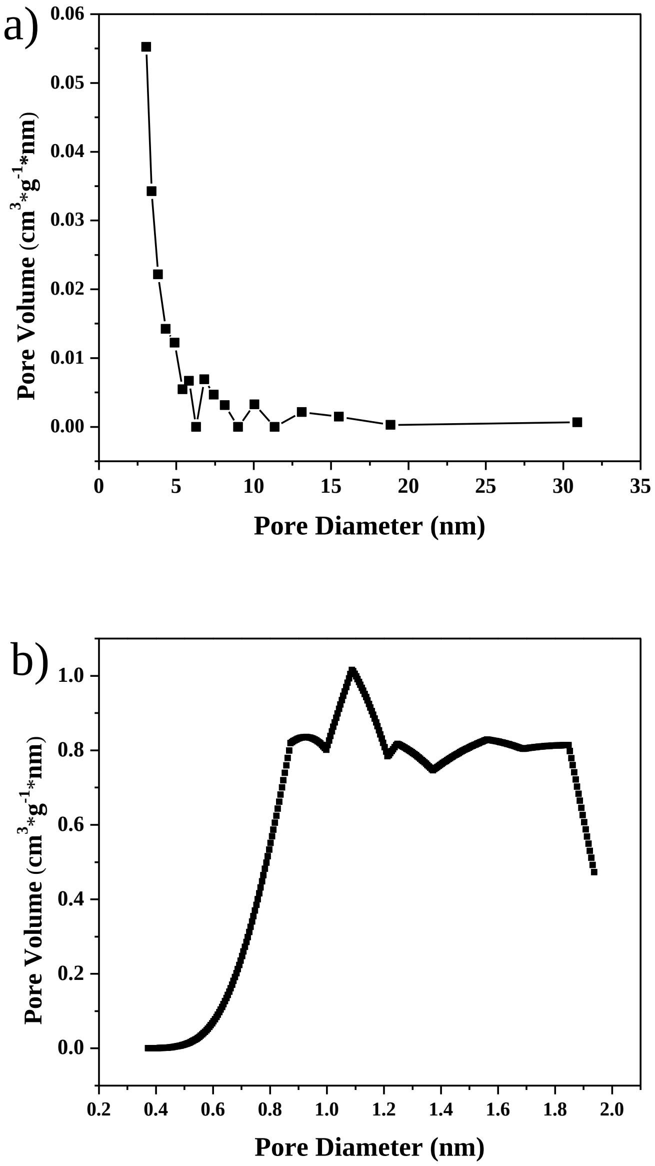

Figure S1. (**a**) The BJH pore size of MIL-101(Cr); (**b**) The HK pore size of MIL-101(Cr).

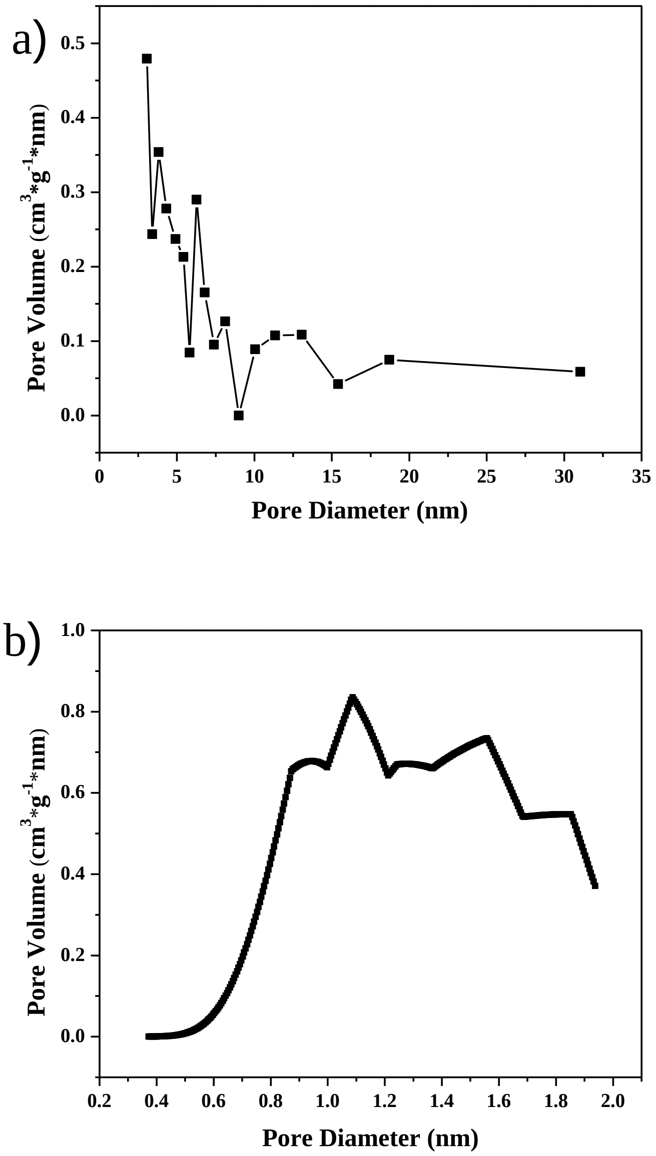

Figure S2. (**a**) The BJH pore size of MIL-101(Cr)-Cl; (**b**) The HK pore size of MIL-101(Cr)-Cl.

Precipitation experiment before and after adsorption

Five milligrams of MIL-101(Cr)-Cl powders were soaked in 10 mL 100 mg/L of 2,4-D for about 12 h; after centrifuged, the supernatants were taken out, and then added 5 mM $AgNO_3$ solution, and 100mg/L of 2,4-D was also added 5 mM $AgNO_3$ solution.

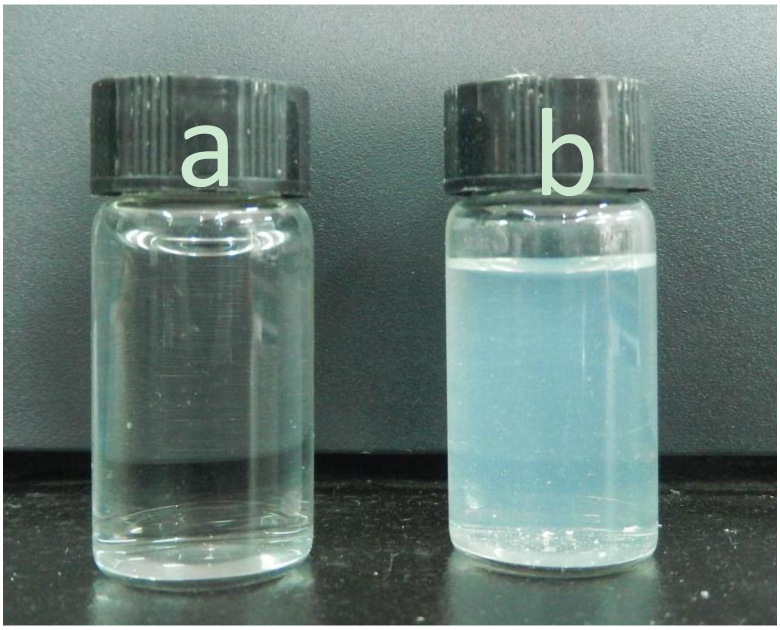

Figure S3. The photos of (**a**) 100mg/L of 2,4-D added $AgNO_3$ solution; (**b**) after adsorbed 2,4-D, the supernatants added $AgNO_3$ solution.

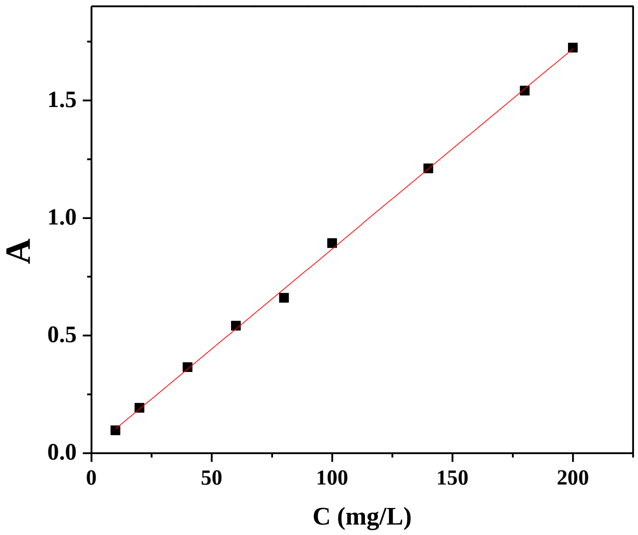

Figure S4. The calibration curve of 2,4-D, $A = 0.0085C+0.0184$, $R^2 = 0.999$.

Table S1. The fluorine and chlorine content in MIL-100-Cr and MIL-100-Cr-Cl.

Sample	Fluorine Content	Chlorine Content
MIL-101(Cr)	5.66 wt %	-
MIL-101(Cr)-Cl	0.04 wt %	10.58 wt %
